# Optimization of the Omni-ATAC protocol to chromatin accessibility profiling in snap-frozen rat adipose and muscle tissues

**DOI:** 10.1016/j.mex.2022.101681

**Published:** 2022-04-01

**Authors:** Venugopalan D. Nair, Mital Vasoya, Vishnu Nair, Gregory R. Smith, Hanna Pincas, Yongchao Ge, Collin M. Douglas, Karyn A. Esser, Stuart C. Sealfon

**Affiliations:** 1 Department of Neurology, Center for Advanced Research on Diagnostic Assays, Icahn School of Medicine at Mount Sinai, New York, NY 10029, USA; 2 Department of Computer Sciences, Columbia University, New York, NY 10027, USA; 3 Department of Physiology and Functional Genomics, University of Florida, Gainesville, FL 32610, USA

**Keywords:** ATAC-seq, Snap-frozen adipose and muscle tissue, Bead-based tissue homogenization, ENCODE quality standards

## Abstract

ATAC-seq is a fast and sensitive method for the epigenomic profiling of open chromatin and for mapping of transcription factor binding sites [1]. Despite the development of the Omni-ATAC protocol for the profiling of chromatin accessibility in frozen tissues [2], studies in adipose tissue have been restricted due to technical challenges including the high lipid content of adipocytes and reproducibility issues between replicates. Here, we provide a modified Omni-ATAC protocol that achieves high data reproducibility in various tissue types from rat, including adipose and muscle tissues [3].

• This protocol describes a methodology that enables chromatin accessibility profiling from snap-frozen rat adipose and muscle tissues.
• The technique comprises an optimized bead-based tissue homogenization process that substitutes to Dounce homogenization, reduces variability in the experimental procedure, and is adaptable to various tissue types.
• In comparison with the Omni-ATAC protocol, the method described here results in improved ATAC-seq data quality that complies with ENCODE quality standards.

## Graphical abstract

**Figure:**
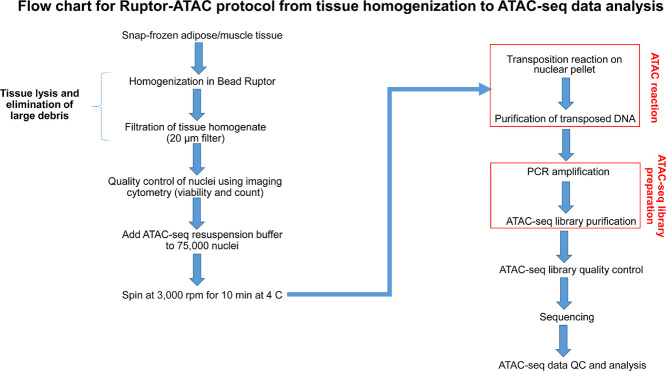

**SPECIFICATIONS TABLE**

**Table:** 

**Subject Area**	Biochemistry, Genetics and Molecular Biology
**More specific subject area**	Assay for Transposase-Accessible Chromatin using sequencing for assaying chromatin accessibility genome-wide
**Method name**	Ruptor-ATAC
**Name and reference of original method**	M Ryan Corces, Alexandro E Trevino, Emily G Hamilton, Peyton G Greenside, Nicholas A Sinnott-Armstrong, Sam Vesuna, Ansuman T Satpathy, Adam J Rubin, Kathleen S Montine, Beijing Wu, Arwa Kathiria, Seung Woo Cho, Maxwell R Mumbach, Ava C Carter, Maya Kasowski, Lisa A Orloff, Viviana I Risca, Anshul Kundaje, Paul A Khavari, Thomas J Montine, William J Greenleaf & Howard Y Chang “An improved ATAC-seq protocol reduces background and enables interrogation of frozen tissues“ Nature Methods volume 14, pages959–962 (2017)
**Resource availability**	N/A

*Method details

## Method Introduction

**Fig. 1:**
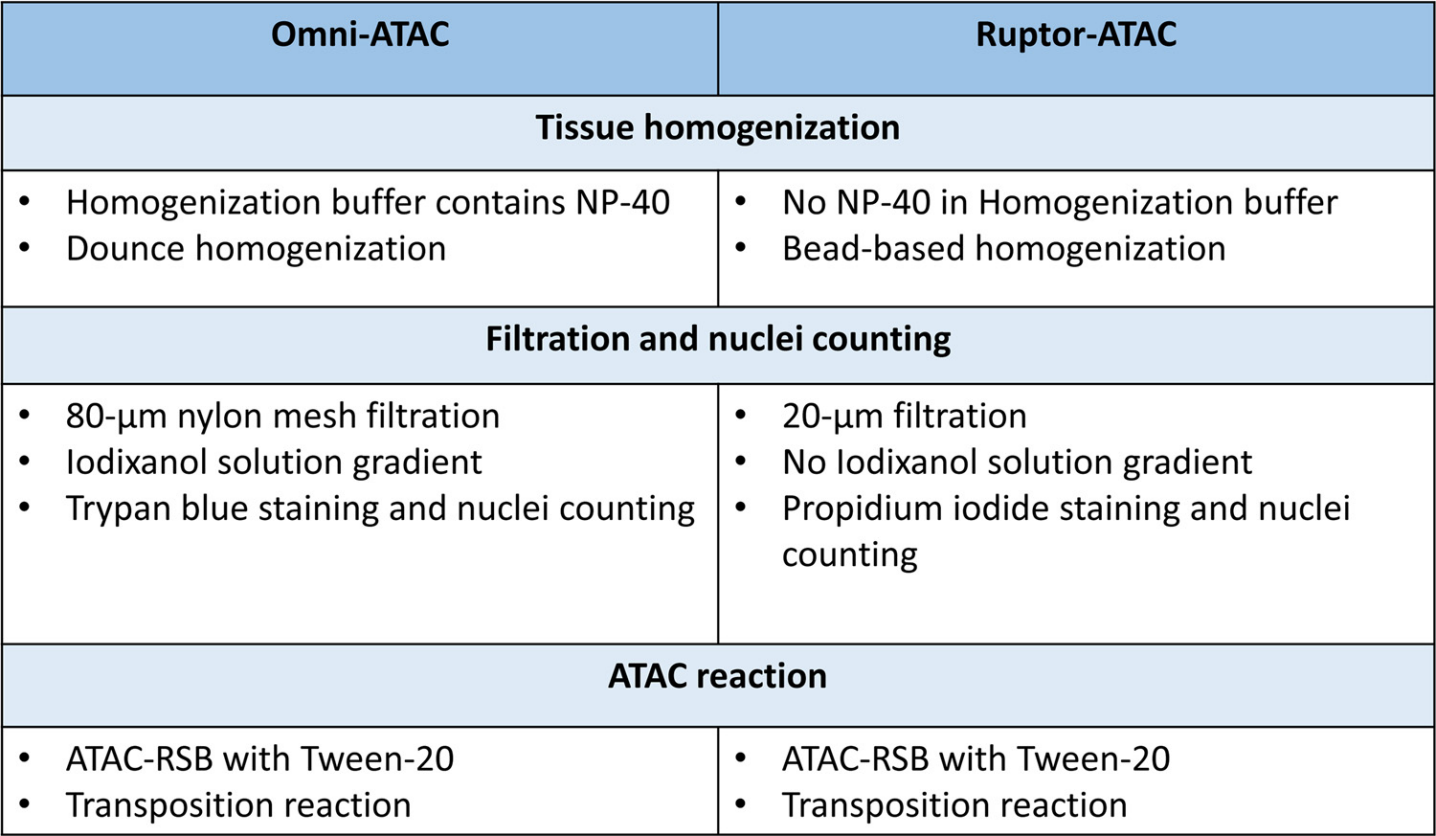
Comparison of the improved ATAC-seq method and the conventional Omni-ATAC method. Table of defined steps in ATAC-seq protocol using the conventional (Omni-ATAC) method versus our optimized protocol (Ruptor-ATAC).

The Assay for Transposase-Accessible Chromatin using sequencing (ATAC-seq), is a low-input, relatively fast and easy-to-perform method, which consequently has been widely used for mapping chromatin accessibility genome-wide and for investigating TF binding [1, 4]. It uses a genetically engineered hyperactive Tn5 transposase that simultaneously cleaves DNA in open chromatin regions and ligates adapters for high-throughput sequencing at these regions [5, 6]. While the Omni-ATAC protocol was developed to profile chromatin accessibility in frozen tissues [2], it has shown a few limitations. Notably, ATAC-seq analyses in adipose tissue or in tissue-derived adipocytes have been scarce, most likely due to the technical challenge posed by the high lipid content of adipocytes [7], [8], [9], [10], [11]. Moreover, low reproducibility between replicates has been observed, with variability in Irreproducible Discovery Rate (IDR) peaks and in transcription start site (TSS) enrichment scores across tissues [9]. In the Omni-ATAC protocol, tissues are homogenized using a Dounce homogenizer, involving a defined number of strokes with the pestles (e.g. 10 with the loose pestle, followed by 20 with the tight pestle), which may differ between tissue types (e.g. soft vs. hard tissues) [12]. Subsequently, the number of isolated nuclei can vary between tissue types, adding some sample-to-sample variability. To optimize the ATAC-seq method to frozen adipose tissue and gain high data reproducibility in various tissues, we modified the Omni-ATAC protocol to include an effective bead-based tissue homogenization process that is applicable to adipose tissue and skeletal muscle. Fig. 1 illustrates the differences between the old and the new protocol. The resulting Ruptor-ATAC protocol is suitable for high throughput studies, complies with the ENCODE quality standards for ATAC-seq data (https://www.encodeproject.org/atac-seq/#standards; [13]), and demonstrates improved ATAC-seq data quality based on metrics such as Usable Reads, IDR peaks, FRiP and TSS enrichment scores.

### Ruptor-ATAC protocol

**Note**: All steps should be performed on ice or at 4°C. Pre-chill a centrifuge to 4°C. Frozen tissue fragments from rat (∼10 mg for muscle and ∼30 mg for adipose tissue) are used in this protocol.

**Required reagents and equipment:**

• 10-ml Micronics V-bottom screw cap tubes (Micronic, USA, Cat.# MP52318)
• Sucrose (Sigma-Aldrich, St. Louis, MO, USA)
• Tween-20, supplied at 10% (Sigma-Aldrich)
• 1M Tris-HCl pH 7.8 buffer (Thermo Fisher Scientific, Waltham, MA)
• 1M Tris-HCl pH 7.4 buffer (Thermo Fisher Scientific)
• 500 mM EDTA (Thermo Fisher Scientific)
• 1M CaCl2 (Thermo Fisher Scientific)
• 1M Mg(Ac)2 (Thermo Fisher Scientific)
• 1M MgCl2 (Thermo Fisher Scientific)
• 5M NaCl (Thermo Fisher Scientific)
• 14.3M β-mercaptoethanol (Sigma-Aldrich)
• Sterile nuclease-free, ultrapure distilled water (H2O; Thermo Fisher Scientific)
• cOmplete™ Protease Inhibitor Cocktail (mini-tablets; Thermo Fisher Scientific, Cat.# A32955)
• 2.8-mm ceramic beads (Omni International, Kennesaw, GA, USA, Cat.# SKU 19-646)
• Bead Ruptor Elite bead mill homogenizer with Omni BR Cryo cooling unit (Omni International, Cat.# SKU 19-042E)
• Liquid nitrogen
• Mini 20-µm pluriStrainer 100/PK (pluriSelect, Leipzig, Germany, Cat.# NC1423042)
• Countess II Automated Cell Counter or Cellometer K2 Image cytometer (Nexcelom, Lawrence, MA)
• Propidium Iodide (PI) dye (Sigma-Aldrich)
• Magnetic Rack (Thermo Fisher Scientific)
• Fixed Angle Centrifuge (Eppendorf 5418R)
• Eppendorf 5418R centrifuge
• 2X TD buffer (Illumina, Inc, San Diego, CA, Cat.# 20034198)
• Tn5 transposase (Illumina, Inc, San Diego, Cat.# 20034198)
• Thermomixer (USA Scientific, Ocala, FL, USA)
• DNA Clean and Concentrator kit - (Zymo Research, Irvine, CA, Cat.# 34014)
• Ethanol
• 0.2-ml PCR tubes
• NEBNext High Fidelity 2x PCR Master mix (New England Biolabs)
• Ad1_noMX PCR Primers (Eurofins Scientific)
• Ad2. x Indexing Primers (Eurofins Scientific)
• KAPA High-Fidelity 2X PCR Master Mix (Sigma-Aldrich)
• PCR thermal cycler
• Agencourt AMPure XP beads (Beckman Coulter, Indianapolis, IN, USA)
• Agilent 2100 Bioanalyzer High-Sensitivity DNA kit (Agilent Technologies, Santa Clara, CA, USA)
• Qubit dsDNA High Sensitivity Assay kit (Thermo Fisher Scientific)
• Novaseq 6000 (Illumina)

Step 1: Tissue homogenization

**Note**: Omni BR Cryo cooling unit is kept at 10°C ahead of time and throughout the tissue homogenization process using liquid nitrogen.

### Stock solution and buffer preparation

Prepare 4x Homogenization Stock Buffer in advance and store at 4⁰C. Sterile filtration is recommended.

4x Homogenization Stock Buffer

**Table:** 

Reagent	Final Concentration	Mass/Volume for 100 ml

Sucrose	1.280 M	43.8 g
1M Tris-HCl pH 7.8	40 mM	4 ml
500 mM EDTA	400 nM	80 ml
1M CaCl2	20 mM	2 ml
1M Mg(Ac)2	12 mM	1.2 ml
14.3M β-mercaptoethanol	668 µM	4.67 µl
H2O	NA	Bring up to 100 ml

### Same day buffer preparation

Prepare 1x Homogenization Buffer on the day of processing. Store at 4⁰C.

1x Homogenization Buffer

**Table:** 

Reagent	Final Concentration	Volume for 10 ml

4x Homogenization Stock Buffer	1x	2.5 ml
Protease Inhibitor Cocktail	NA	1 mini-tablet
H2O	NA	Bring up to 10 ml

1. Transfer frozen tissue sample to a 1.1-ml micronic tube and add in 600 µl of cold 1x Homogenization Buffer. Resuspend by pipetting up and down.
2. Add three, 2.8-mm ceramic beads to the micronic tube.
3. Vortex micronic tube thrice for 5-10 seconds and keep it on ice.
4. Homogenize tissue sample in a Bead Ruptor Elite bead mill homogenizer with Omni BR Cryo cooling unit using 2 cycles of the following settings: speed: 1.0, time: 20 sec and speed: 2.1, time: 20 sec, dwell time: 20 sec at both speeds.

Step 2: Homogenate filtration and nuclei counting

1. Following homogenization, filter tissue homogenate through a mini 20-µm pluriStrainer. Wait for 20-30 sec.
2. Count nuclei in the filtrate using an Automated Cell Counter such as Countess II or Cellometer K2 Image cytometer according to the manufacturer's instructions.
4. Aliquot nuclei for ATAC reaction.

**Note**: If the tissue homogenate does not pass through the strainer, vortex it quickly to let it pass. Adipose tissue homogenates do not pass easily through the strainer even after spinning. For such homogenates, use another strainer to filter.

Step 3: ATAC reaction

### Same day buffer preparation

ATAC-seq Resuspension Buffer (ATAC-RSB; as described in [2] 28846090) containing 0.1% Tween-20

**Table:** 

Reagent	Final Concentration	Volume for 50 ml

1M Tris-HCl pH 7.4	10 mM	500 µl
5M NaCl	10 mM	100 µl
1M MgCl2	3 mM	150 µl
10% Tween-20	0.1%	500 µl
H2O	NA	49.75 ml

Transposition reaction mixture

**Table:** 

Reagent	Fold Dilution (x)	Volume for 50 µl

2x TD buffer	2x	25 µl
PBS	3x	16.7 µl
1% digitonin	100x	0.5 µl
10% Tween-20	100x	0.5 µl
Tn5 transposase	33.33x	1.5 µl
H2O	NA	5.8 µl

1. Transfer approximately 75,000 nuclei into a fresh 2.0 ml Eppendorf tube and dilute them in 1 ml of cold ATAC-RSB containing 0.1% Tween-20. Mix by inverting tube 10 times.
2. Centrifuge nuclei for 10 min at 3,000 rpm at 4°C in an Eppendorf 5418R centrifuge.
3. Carefully aspirate supernatant (aspirate down to 100 µl with a p1000 pipette and remove final 100 µl with a p200 pipette).
4. Add 50 µl transposition reaction mixture to the nuclear pellet and resuspend by pipetting up and down 6 times.
5. Incubate transposition reaction at 37°C for 30 min using a thermomixer with 1,000 rpm mixing.
6. Immediately transfer to ice after Incubation.
7. Purify transposed DNA using a DNA clean and concentrator kit as follows:
  7.1. In a 1.5-ml Eppendorf tube, add 5 volumes (250 µl) of DNA binding buffer to one volume of DNA sample (50 µl). Mix briefly by vortexing.
  7.2 Transfer mixture to Zymo-Spin Column in a collection tube. Centrifuge for 1 min at 8,000 rpm. Discard the flow-through.
  7.3 Add 300 µl of DNA wash buffer to the column. Centrifuge 1 min. Discard the flow-through. Repeat this step and centrifuge for 1 min at 8,000 rpm.
  7.4 Add 20 µl/22 µl of Elution Buffer or Ultrapure Distilled Water directly to the column matrix and incubate at room temperature for 1 min.
  7.5 Transfer the column to a 1.5-ml Eppendorf tube and spin for 1 min to elute the purified, transposed DNA.
8. Store at −20°C if necessary.

Step 4: ATAC-seq library preparation

1. PCR amplify purified, transposed DNA fragments by combining the following in a 0.2-ml PCR tube:
  20 μl transposed DNA
  1.0 μl nuclease-free H2O
  2.0 μl Ad1_noMX primer (25 µM)
  2.0 μl Ad2. *Indexing Primer (25 µM)
  25 μl KAPA High-Fidelity 2X PCR Master Mix
  Thermal cycle as follows:
  1 cycle: 72°C for 5 min; 98°C for 30 sec
  10-11 cycles: 98⁰C for 10 sec, 63⁰C for 30 sec, 72⁰C for 1 min
2. Following amplification, transfer PCR tubes on ice or at 4⁰C to stop the reaction.
3. Purify PCR amplified library using Agencourt AMPure XP beads: perform a double-sided bead purification (to remove primer dimers and large >1,000 bp fragments) by following the steps described on the Kaestner Lab website under Resources Protocols (https://www.med.upenn.edu/kaestnerlab/protocols.html, **UPDATED** ATAC-seq Protocol - January 28, 2019)
4. Elute the purified library in 20 μl elution nuclease-free water buffer. Ensure that the column is dry prior to adding elution buffer to avoid ethanol contamination in the final library.
5. Store the purified libraries at −20°C if necessary.

Step 5: Library pooling and sequencing

**Note:** Prior to pooling, libraries are quantified and their quality is evaluated (see Method validation).

1. Pool all the purified libraries.
2. Sequence the pooled libraries using 100-bp paired-end reads on the Illumina Novaseq 6000.

## Method validation

### ATAC-seq library quality control

1. Determine the quantity of purified libraries using the Qubit dsDNA high sensitivity assay kit, which provides the DNA concentration measured on the fluorometer.
2. Assess the quality of purified libraries using an Agilent Bioanalyzer High-Sensitivity DNA kit, which gives information about the size distribution.

**Fig. 2:**
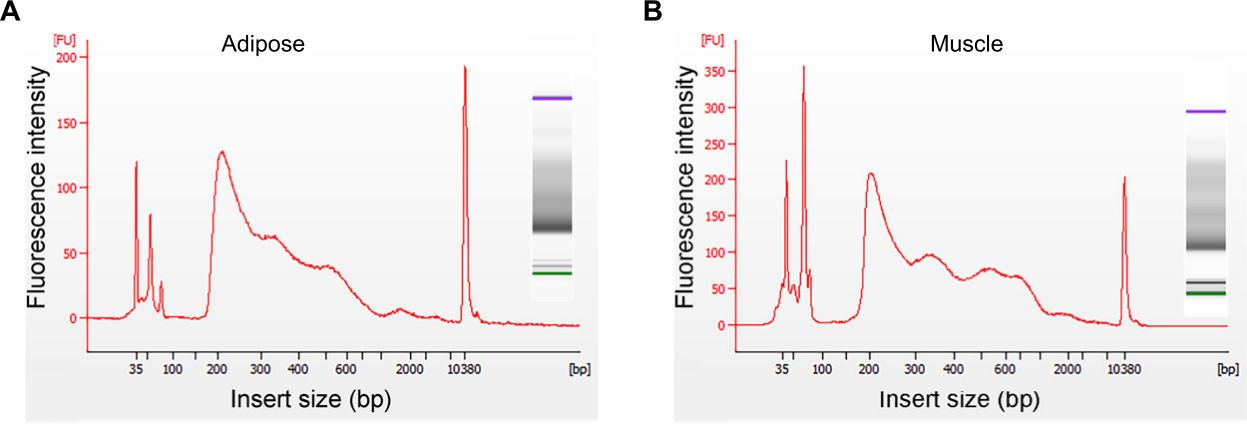
Quality control of ATAC-seq libraries. Fragment sizes for two representative ATAC-seq libraries, determined by Bioanalyzer. A size distribution plot and a gel electrophoresis-like image are presented for an adipose sample (**A**) and a muscle sample (**B**). Two molecular weight markers are used: a 35-bp and a 10,000-bp marker, which appear in green and in purple, respectively, in the gel-like image. Adapted from [3].

The nucleosome is comprised of a histone octamer that is complexed with approximately 147 bp of DNA [14]. Adapters are an additional 142 bp. A typical fragment size distribution plot shows an enrichment in nucleosome-free fragments (∼200 bp) and mono-nucleosome-bound fragments (∼300 bp), followed by di-nucleosome-associated regions (∼500 bp). Both adipose and muscle ATAC-seq libraries show the expected fragment size and nucleosome phasing (Fig. 2).

### ATAC-seq data analysis and quality control

Required software:

• ENCODE ATAC-seq pipeline v1.7.0 (https://www.encodeproject.org/atac-seq/)
• FastQC [15]
• Trimmomatic [16]
• Bowtie2 [17]
• Picard tools (https://broadinstitute.github.io/picard/)
• MACS2 [18]
• HOMER
• Integrated Genomic Viewer (IGV 2.3.61)
• DiffBind package (v1.2.4) [19]

1. Process the ATAC-seq data (trim, align, filter, and quality control the data) using the ENCODE ATAC-seq pipeline v1.7.0. Table 1 presents the ATAC-seq metadata and mapping statistics obtained using the Ruptor-ATAC protocol, along with the ATAC-seq metrics previously acquired by Liu et al. using the Omni-ATAC protocol [9]. Reproducible peaks range from 44,673 to 54,458 in adipose, and from 84,195 to 112,272 in muscle samples (IDR peaks). TSS enrichment values are generally well above the cutoff (>9; https://www.encodeproject.org/atac-seq/#standards), and the fraction of reads in called peak regions (FRiP) scores are mostly greater than 0.2, denoting the high quality of the ATAC-seq data.

**Table 1:** Comparison of the ATAC-seq data quality metrics obtained using Ruptor-ATAC (see in [3]) vs. those previously collected in mouse adipose and skeletal muscle tissue using Omni-ATAC [9]. Mapped reads, total number of reads minus number of unaligned reads. chrM reads, mitochondrial chromosome reads. Usable reads, number of mapped reads minus number of low mapping quality, duplicate, and mitochondrial reads. % Usable reads, Usable reads to Total reads ratio. TSS enrichment, TSS enrichment score. IDR Peaks, Irreproducible Discovery Rate on more than two replicates. FRiP, Fraction of reads in peaks. N.a., not available.

Ruptor-ATAC protocol													
Sample ID	Gender	Total reads	Mapped reads	chrM Reads	Usable Reads	% of Usable Reads	AVERAGE % of Usable Reads	TSS Enrichment	AVERAGE TSS Enrichment	Number of Peaks	IDR Peaks	AVERAGE IDR Peaks	FRiP
Adipose-1	Male	186920128	152454036	3325180	152247883	81.5	90.9	11.31	11.18	131709	52307	48311	0.21
Adipose-2	Male	154260126	147601827	2934811	145736711	94.5		11.92		134503	53745		0.22
Adipose-3	Male	171080622	160714303	2968216	158805941	92.8		11.12		166811	46164		0.19
Adipose-4	Male	151594180	144181953	3878928	141673087	93.5		12.29		161960	54458		0.23
Adipose-5	Male	143599246	134322230	2566141	132644041	92.3		9.25		169572	34883		0.18
Muscle-1	Male	181961570	168227640	2542107	166550226	91.5	90.1	16.64	20.75	207168	92617	98196	0.40
Muscle-2	Male	239434514	216391974	3753295	213924262	89.3		18.52		200610	94235		0.41
Muscle-3	Male	196768302	176279821	3257512	174089894	88.5		17.63		207911	84195		0.41
Muscle-4	Male	200874760	185106070	5941583	181097830	90.2		25.18		208890	107660		0.52
Muscle-5	Male	249010484	229770406	5103892	226359211	90.9		25.78		208580	112272		0.52
**Omni-ATAC protocol (data from Liu et al., 2019 [****9****])**													
ATAC-7 (Adipose)	Female	170643536	166598249	1204915	61090112	35.8	36.4	13.03	12.83	n.a.	28827	28827	n.a.
ATAC-8 (Adipose)	Female	165896236	135431464	1175420	61493964	37.1		12.63		n.a.	28827		n.a.
ATAC-59 (Muscle)	Male	117557552	115960450	1920255	44835398	38.1	29.0	6.32	7.92	n.a.	14722	14722	n.a.
ATAC-60 (Muscle)	Male	214824060	211626739	2469962	42681444	19.9		9.52		n.a.	14722		n.a.
2. For all samples, read quality is assessed using FastQC.
3. Trimmomatic is used to remove adapters and low-quality base pairs and reads are identified by FastQC.
4. Reads for each sample are aligned to the rat genome (rn6.0) using Bowtie2.
5. After alignment, reads mapping to the mitochondrial genome are removed.
6. As a general measure of sensitivity to Tn5 transposase fragmentation, ATAC-seq signal is defined as number of transposase cuts mapping to each bp (or a total count of cuts mapping to a selected genomic interval). The cuts are defined as 5’ ends of ATAC-seq reads, with additional shifting by +4 bp and -5 bp for reads mapping to the plus and minus strands, respectively [4] (Buenrostro 2013). Duplications are removed by Picard tools.
7. MACS2 is applied on each merged bam file to call peaks (with option –nomodel –extsize 200 –shift 100).
8. HOMER is used to annotate ATAC-seq peaks to the various genomic regions (promoter, 5′ UTR, exon, intron, 3′ UTR, downstream, and intergenic), and assign them to the nearest gene based on rat genome assembly rn6. Notably, promoters are defined as 3 kb upstream and downstream from the TSS. Downstream regions correspond to the 3 kb DNA region located downstream from the transcription termination site.
9. All sequencing tracks, bigWig files are viewed using the Integrated Genomic Viewer. Fold-change of ATAC-seq signal is calculated using DiffBind package in R. Interpretation is limited to peaks that exceed a L2FC of 1 (FDR < 0.01) in either direction.

## Declaration of Competing Interests

The authors declare that they have no known competing financial interests or personal relationships that could have appeared to influence the work reported in this paper.
